# Correlative Super-Resolution Optical and Atomic Force Microscopy Reveals Relationships Between Bacterial Cell Wall Architecture and Synthesis in *Bacillus subtilis*

**DOI:** 10.1021/acsnano.1c04375

**Published:** 2021-09-17

**Authors:** Raveen K. G. Tank, Victoria A. Lund, Sandip Kumar, Robert D. Turner, Lucia Lafage, Laia Pasquina Lemonche, Per A. Bullough, Ashley Cadby, Simon J. Foster, Jamie K. Hobbs

**Affiliations:** † Department of Physics and Astronomy, University of Sheffield, Sheffield S3 7RH, United Kingdom; § Department of Molecular Biology and Biotechnology, University of Sheffield, Sheffield S10 2TN, United Kingdom; ∥ The Florey Institute for Host−Pathogen Interactions, University of Sheffield, Sheffield S10 2TN, United Kingdom; ⊥ Department of Biochemistry, University of Oxford, Oxford OX1 3QU, United Kingdom; ‡ Department of Computer Science, University of Sheffield, Sheffield, S1 4DP, United Kingdom

**Keywords:** atomic force microscopy, stochastic optical reconstruction microscopy, structured illumination microscopy, correlative microscopy, super-resolution, bacterial growth, peptidoglycan

## Abstract

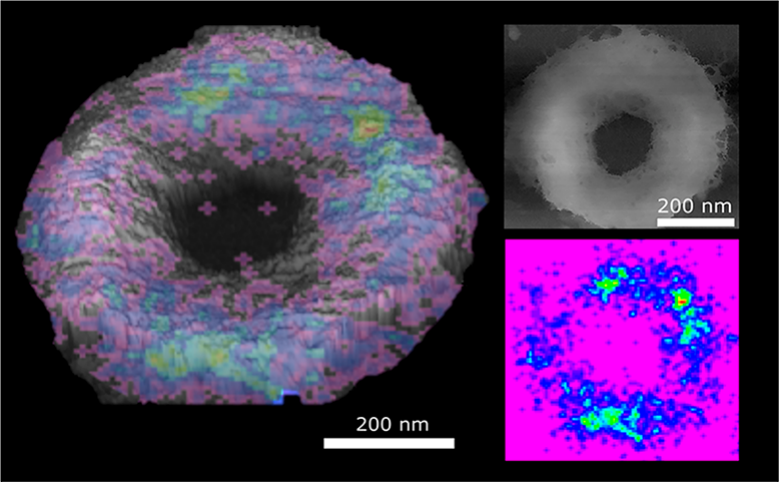

Understanding how bacteria grow and divide requires insight into both the molecular-level dynamics of ultrastructure and the chemistry of the constituent components. Atomic force microscopy (AFM) can provide near molecular resolution images of biological systems but typically provides limited chemical information. Conversely, while super-resolution optical microscopy allows localization of particular molecules and chemistries, information on the molecular context is difficult to obtain. Here, we combine these approaches into STORMForce (stochastic optical reconstruction with atomic force microscopy) and the complementary SIMForce (structured illumination with atomic force microscopy), to map the synthesis of the bacterial cell wall structural macromolecule, peptidoglycan, during growth and division in the rod-shaped bacterium *Bacillus subtilis*. Using “clickable” d-amino acid incorporation, we fluorescently label and spatially localize a short and controlled period of peptidoglycan synthesis and correlate this information with high-resolution AFM of the resulting architecture. During division, septal synthesis occurs across its developing surface, suggesting a two-stage process with incorporation at the leading edge and with considerable in-filling behind. During growth, the elongation of the rod occurs through bands of synthesis, spaced by ∼300 nm, and corresponds to denser regions of the internal cell wall as revealed by AFM. Combining super-resolution optics and AFM can provide insights into the synthesis processes that produce the complex architectures of bacterial structural biopolymers.

The primary structural component of the Gram-positive bacterial cell wall is peptidoglycan, a macromolecule of relatively stiff glycan chains cross-linked by flexible peptide bridges.^1^ Peptidoglycan governs cell shape, provides the mechanical constraint for cellular osmotic (turgor) pressure, and acts as the scaffold supporting numerous proteins and polymers that interact with the environment, and its synthesis is the target for major antibiotics.^2^ Understanding how cell wall peptidoglycan governs bacterial shape, growth, and division necessitates not only resolution of its molecular architecture but also insight into how it retains the ability to reorganize as a single cross-linked macromolecule in the face of internal turgor pressure. Very recent work has used atomic force microscopy (AFM) to image the peptidoglycan architecture, which in the model rod-shaped organism *Bacillus subtilis* is found to consist of an open porous mesh on the external surface of the cylinder, approximately circumferentially oriented strands on the internal cylinder surface typically spaced by <6 nm, dense rings on the external poles, and a random dense mesh on the internal poles.^3^ Intriguingly, the internal cytoplasm facing surface of the septum that forms during cell division is found to be dense and randomly organized, in contrast to the circumferential rings on its external surface, but with large pores which must be filled in prior to completion of division to avoid cell lysis.

The synthesis of peptidoglycan requires multiple interacting components. *B. subtilis*, like many rod-shaped bacteria, has two groups of proteins that coordinate synthesis, with partially interchangeable components, the so-called “divisome” (machinery for septation), responsible for synthesizing the septum of cell wall that divides a mother cell into two daughters, and the “elongasome” (machinery for growth in the rod/sidewall), responsible for increasing the length of the cell.^4−6^ These “multi-molecular machines” are, in part, coordinated by the filament forming proteins FtsZ and MreB, respectively.^7−9^ Fluorescence microscopy has been used extensively to explore cell wall synthesis, and the development of fluorescent d-amino acids (FDAAs), which are incorporated into peptidoglycan by bacterial peptidoglycan synthesis enzymes, allows localization of nascent material in the context of the whole cell.^10−12^ To date, this approach has revealed the deposition of PG at discrete sites at the leading edge of the septum, associated with the “treadmilling” behavior of FtsZ filaments around the septal ring in *B. subtilis.*(7) In contrast, in the spheroid bacterium *Staphylococcus aureus*, the septal synthesis occurs across its surface as well as around the cell periphery.^13^

Although super-resolution microscopy allows single molecule localization of components of the cell wall synthesis machinery and the time-labeled location of new material within the context of the whole cell, it supplies limited information on the local molecular organization of new material or how it fits into the pre-existing architecture of the cell wall itself. AFM, in contrast, cannot elucidate when or where the new cell wall is deposited, simply revealing cell wall topography. Combining AFM with super-resolution microscopy gives an enhanced perspective of both chemistry and architecture.^14,15^ Here, we use super-resolution fluorescence to locate recent areas of cell wall synthesis, within the architectural context provided by AFM, leading to insights into the processes of both cell division and growth.

## Results/Discussion

### Correlative AFM and Super-Resolution Optical Microscopy: STORMForce and SIMForce

**Figure 1:**
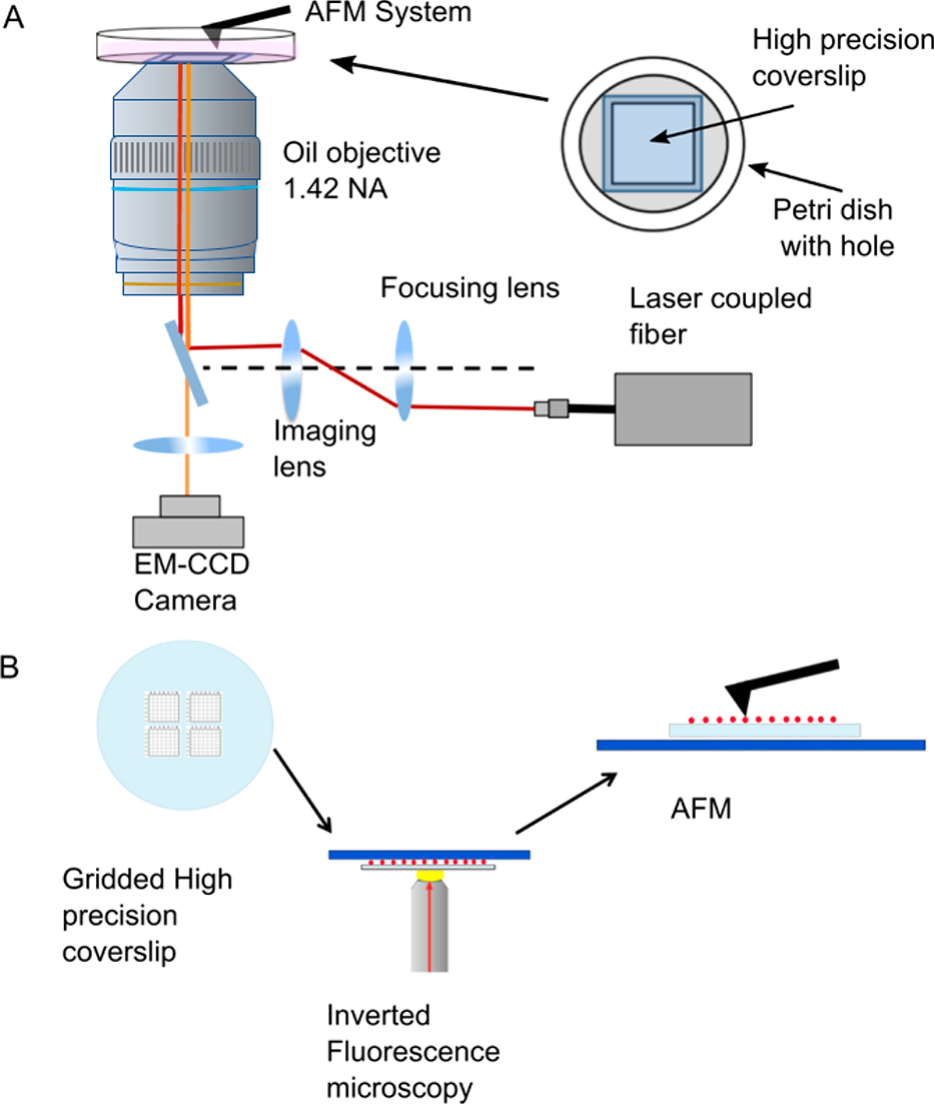
Microscopy setup. (A) Integrated STORMForce setup identifying the addition of the laser and EM-CCD camera, along with the laser light path to (red), and from (orange), the sample. The sample was imaged on a high-precision coverslip mounted over a hole within a Petri dish to hold imaging buffer. The top surface of the buffer was open to the air. (B) Setup of correlative STORMForce or SIMForce where gridded coverslips were used to enable correlated fluorescence imaging and AFM on separate equipment.

Stochastic optical reconstruction microscopy (STORM) provides a robust method based on single molecule localization to obtain super-resolution optical fluorescence images with sub-50 nm resolution.^16^ We converted the inverted optical microscope beneath a JPK Nanowizard III AFM into a STORM through the addition of a 70 mW 642 nm laser as a light source, routed to the microscope *via* an optical fiber to remove noise associated with open optics that may couple to the AFM, and an EM-CCD Hamamatsu camera to collect the single molecule optical signal associated with stochastic blinking of fluorophores (Figure 1A and Methods). Simultaneous AFM and STORM imaging is problematic due to the high optical intensity associated with STORM and concomitant sample heating and hence imaging noise, so imaging with the different modalities was carried out sequentially. The upper surface of the imaging buffer was left open to the air with the lower surface of the sample stuck down to the sample stage and not moved in between imaging modalities. A STORM image was acquired first to identify cell walls (sacculi) and regions of interest. The AFM cantilever was then lined up to these chosen features, the EM CCD camera and imaging laser were turned off, STORM buffer was swapped with AFM imaging buffer, and then AFM imaging commenced. Image overlay was initially performed through use of fiducial markers (see Methods), but it was found that in samples with relatively dense sample dispersion, alignment was possible by direct overlay (“dead reckoning” based on colocation of the two imaging modalities) and adjustment to ensure good correlation across the resulting STORMForce image.

STORM can give very high-resolution optical images, with single molecule localization, but remains a relatively slow technique and can be prone to artifacts associated with the localization software. To confirm data obtained with STORMForce, and to improve throughput, we also developed an approach for correlative structured illumination microscopy (SIM) with AFM (SIMForce). SIM optics are complex, so rather than incorporating them into the same instrument, we instead used a simple finder grid approach in which a sample was first imaged with the SIM and then the same area located on the AFM (Figure 1B and Methods).

### Peptidoglycan Synthesis during Growth and Division

**Figure 2:**
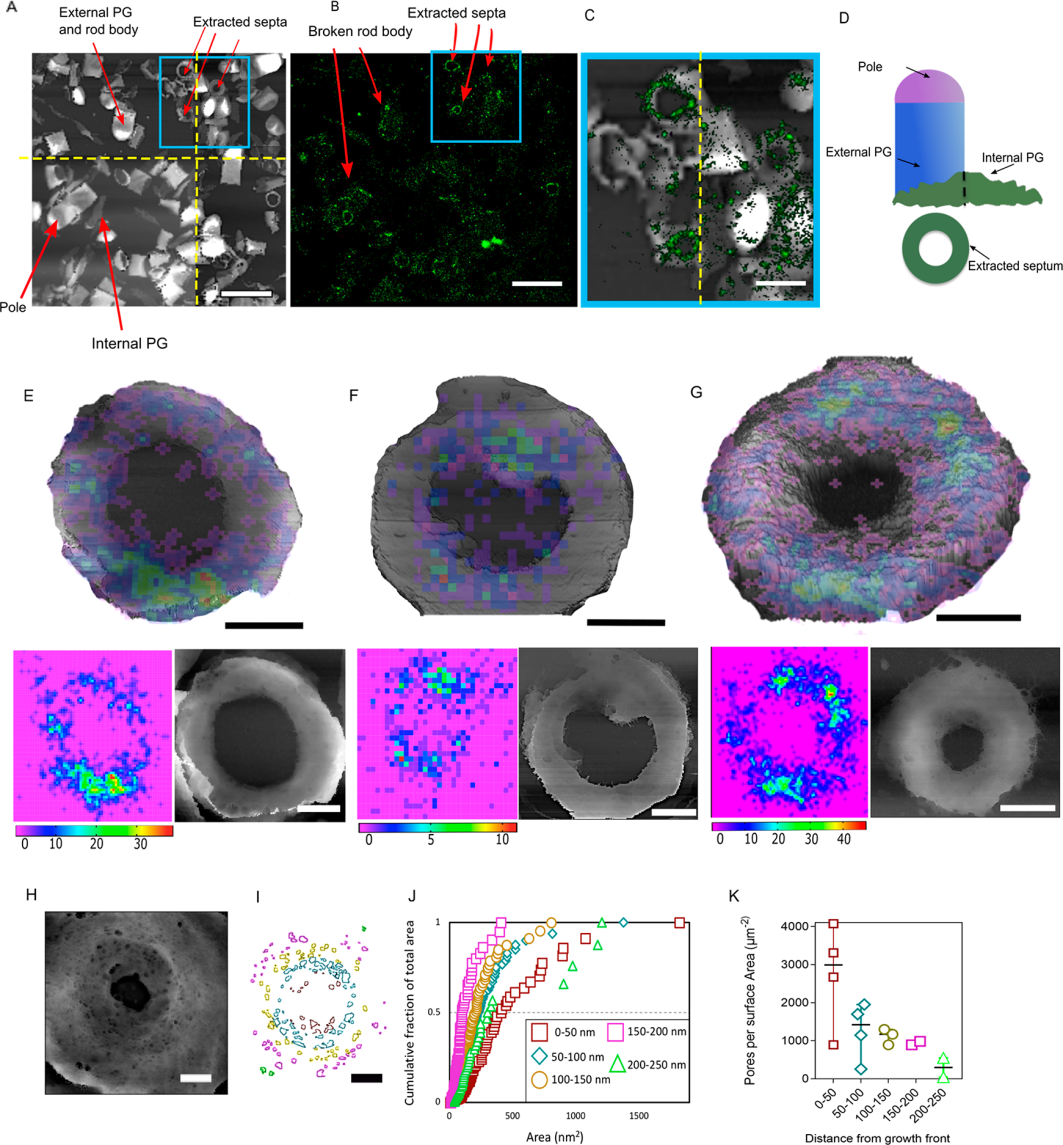
Peptidoglycan insertion at the septum in *B. subtilis*. (A) Tiled AFM images (yellow dotted lines show the borders between images) where arrows indicate regions of interest in a sample of broken sacculi (scale bar 2 μm). Height color scale, 636 nm. (B) STORM image of the same area of sacculi as in (A), with key features annotated (scale bar, 2 μm). (C) The boxed region in (A) and (B) indicates the area of the STORMForce image shown here. The yellow dotted line shows the border between two adjacent AFM images (scale bar, 1 μm). (D) Schematic of sacculi with key features annotated. (E, F) STORMForce imaging of 15 s ADA-labeled septa showing all localizations. Height color scales, (E) 552 nm and (F) 383 nm. The overlay consists of a rainbow STORM localization image placed on top of a 3D AFM image of the same septum. The red on the color scale represents areas of high intensity localizations, indigo areas of low intensity localizations, and violet shows areas where there are no localizations; hence, violet is not shown on the overlaid images. The STORM intensities have been rebinned in (F) to improve signal-to-noise, hence the larger pixel size. Below each image are the separate STORM and AFM topography images (scale bars, 200 nm). (G) As in (E, F) but for a 2 min labeled septum (scale bar, 200 nm; height scale, 472 nm). (H) Example AFM image of a septum and (I) associated pore distribution map; scale bar, 200 nm; height scale, 229 nm. (J) Cumulative fraction of total pore area plotted against the area of each pore in 50 nm circumferential sections of the septa from zero at the leading edge. (K) Number of pores per unit surface area in each 50 nm section of the septa from the leading edge. Each data point is a separate analyzed septum.

As cell wall synthesis occurs at the interface between existing peptidoglycan and the plasma membrane, inaccessible in live cells to the AFM tip, we focused our attention on extracted sacculi which were gently broken to reveal internal and external surfaces and developing septa. The cell wall was labeled by incorporation of FDAAs, introduced into the bacterial culture for specific times prior to harvesting. This labeling indicates the location of newly incorporated peptides in the peptidoglycan.^17^ Single FDAAs (ADA) can be inserted into peptidoglycan *via* both synthesis and exchange reactions, while dipeptides (ADA-DA) can be inserted *via* synthesis only.^11^ In the AFM, sacculi fragments are visualized as flattened, cell-shaped objects, approximately 60 nm thick when imaged in liquid, 1.5 μm wide, and several micrometres long depending on their stage in the cell cycle.^3^ Partially and fully formed septa appear as rings and disks respectively (Figure 2A,D). In fluorescent FDAA-labeled samples, the optical images show a similar overall sacculus morphology, but with higher fluorescence intensity at the septal region, whereas the cell cylinder has areas of variable labeling (Figure 2 B).

### Peptidoglycan Synthesis during Septation

AFM reveals the developing septum as a disk with a central annulus.^3^ The incomplete septum has large pores (>10 nm diameter) behind the leading edge, whereas the internal surface of poles (which septa become, following division) consist entirely of a dense mesh without large pores.^3^ The observed peptidoglycan architectures therefore suggest septal biogenesis as at least a two-step process with initial synthesis to create the closing aperture and then backfilling of pores. FDAA incorporation was used to map nascent peptidoglycan synthesis onto the developing septal structure as revealed by AFM. A 15 s FDAA labeling time was used, as this method has previously been shown to allow the molecular pattern of synthesis to be determined.^13^ Both SIM and STORM revealed a non-uniform FDAA incorporation pattern, similar for both ADA and ADA-DA (Figure 2, SI Figures S1 and S2). SIM demonstrates a primary intensity of labeling in an uneven ring from the leading edge and across the septum, which became more pronounced with longer incorporation periods (Figure S1). However, the level of resolution obtained by this approach does not permit further details to be determined. Molecular localization from STORM imaging also allowed the localization of nascent peptidoglycan synthesis to be elucidated with 15 s labeling (Figure S2A). Synthesis occurred across the septum, with some aggregations of labeling.

Acquiring AFM and STORM data for the same sample, STORMForce reveals where nascent synthesis has occurred against the background of the septal architecture. The distribution of FDAA localizations shows significant intensity over the entire width of the septum, regardless of aperture size (Figure 2 E,F and Figure S2). While not evenly distributed, there is limited accumulation of synthesis at the leading edge, some apparent aggregations occur across the septum and are not regular in number or distribution between septa. Significant fluorescence intensity is also seen away from the aggregations, implying continued synthesis across the septum. A longer labeling time of 2 min also did not reveal a distinct pattern of synthesis, but rather that aggregations of labeling became larger and there was more general labeling across the septum (Figure 2G and Figure S2).

In *B. subtilis*, septa within sacculi samples consist of the nascent poles of both daughter cells and are hence approximately twice as thick as the rest of the cell wall. It is not possible to differentiate between the fluorescent signal coming from the top and bottom surface, so we cannot directly correlate the material observed under the AFM with most recent growth. To explore the profile of the growing septa, we therefore used transmission electron microscopy (TEM), observing sections of the *B. subtilis* septa through the cell cycle. Unlike *S. aureus*, which exhibits “V-shaped” septa,^13^ TEM of *B. subtilis* shows an approximately uniform septal thickness from leading to lagging edge, following the initiation of division, characterized by an angle of ∼90° at the septum–rod interface throughout septation (Figure S3). This precludes an identical model for septal synthesis to that found in *S. aureus*, where the initially bevelled septum increases the available surface area and permits rapid synthesis,^13^ and creates a question as to how synthesis across the developing septal plate can occur in a septum that has parallel sides and does not increase in thickness behind its leading edge. A possibility is that the pores observed in unfinished septa^3^ may provide a template with the potential for subsequent in-filling. To further explore this suggestion, we calculated the size and density of peptidoglycan pores with respect to the distance from the leading edge of the septa from our AFM data (Figure 2 H–J). There are large pores in the peptidoglycan existing at all distances from the septal leading edge right back to the interface of the septum with the cylinder. As the septal annulus is filled, there is a wide range of pore sizes from the leading edge (Figure 2J). The percentage of area covered by pores reduces further away from the leading edge (Figure 2K). These observations imply that in-filling of pores happens randomly, that is, large pores are not preferentially filled as otherwise we would observe fewer large pores at larger radii (older regions of the septum), but that in-filling occurs across the septum before the annulus is closed. The high level of porosity of the septa in *B. subtilis* contrasts with previous AFM data of the coccoid bacterium *S. aureus* in which the septa are uniformly dense behind the leading edge,^3^ albeit with steadily reducing thickness toward the central aperture.

Collectively, these data suggest that septal synthesis occurs as at least a two-stage process. The first involves deposition of new cell wall at the leading edge of the septum; this will allow the closing of the septal annulus. As this material is laid down, it is not in its mature form, containing partially finished regions of overly loose mesh or even large pores. Some of these pores are sufficiently large and deep that they would threaten the integrity of the cell wall if the bacteria were to complete division and separation with them still in place. The next stage of synthesis is the largely stochastic infilling of these pores to provide the final uniform septum ready for cell division. It may be that this mechanism reveals a greater surface area for peptidoglycan insertion during division, allowing the septum to form more rapidly than it could if completed during a single stage at the leading edge.

### Peptidoglycan Synthesis during Elongation

**Figure 3:**
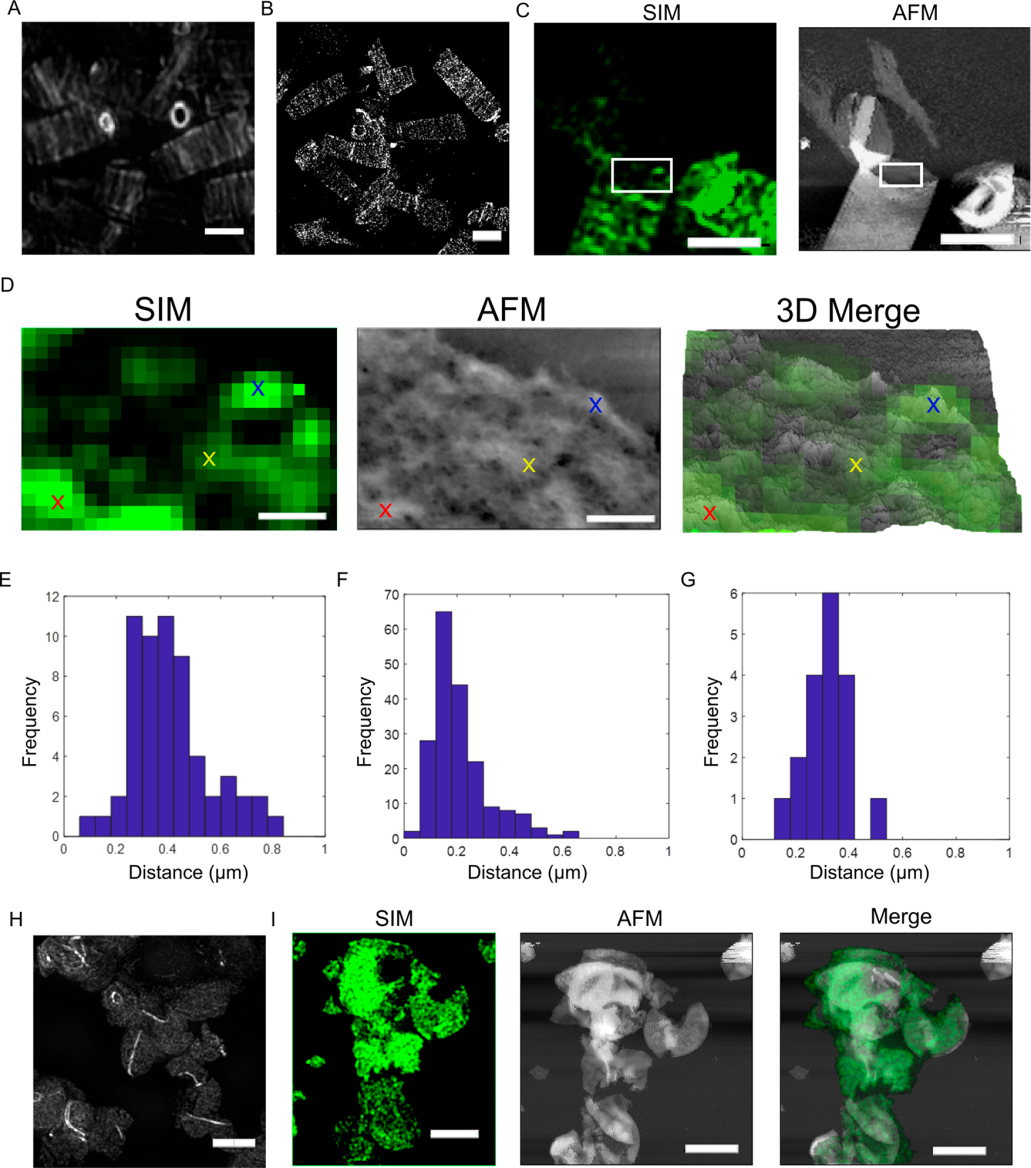
Cylinder peptidoglycan synthesis in *B. subtilis*. (A) SIM image of 15s ADA-DA-labeled *B. subtilis* sacculi (scale bar, 1 μm). (B) STORM image of 15 s ADA-DA-labeled *B. subtilis* sacculi (scale bar, 1 μm). (C) SIMForce of 15 s ADA-DA-labeled *B. subtilis* sacculi (scale bar, 1 μm). (D) SIMForce of the boxed region in (C) showing a single layer of cell wall with 15 s ADA-DA labeling. Colored crosses indicate which areas are correlated for the overlay in the 3D merge (scale bar, 100 nm). (E) Distances between stripes for 15 s labeled sacculi (two layers of cell wall) from SIM images. (F) Distances between stripes from 15 s labeled sacculi (two layers of cell wall) from STORM images. (G) Distances between stripes from 15 s labeled sacculi (single layer of cell wall) from SIM images. (H) SIM image of 15 s ADA-DA-labeled *mreB mbl mreBH* (4577, *Ωneo3427 ΔmreB Δmbl::cat ΔmreBH::erm Ω(neo::spc ΔrsgI*) (scale bar, 5 μm). (I) SIMForce of *mreB mbl mreBH* (scale bar, 2 μm).

In the rod-shaped *B. subtilis*, elongation occurs *via* peptidoglycan synthesis along the cell cylinder.^18^ Synthesis involves the activity of two sets of components. The class A penicillin binding proteins (PBPs) make peptidoglycan, whereas the class B PBPs cross-link glycans polymerized by another component called RodA.^19,20^ The Class B PBPs and RodA form part of the Rod complex, which is responsible for the rod shape and moves around the cell circumference interacting with the Mre components.^21,22^ In order to determine how the cell cylinder is synthesized, a combination of super-resolution fluorescence microscopy and AFM was used (Figure 3). FDAA labeling of whole cells for 15 s to 10 min, followed by purification of sacculi revealed PG synthesis along the cell cylinder as well as at the septum (Figure 3, Figures S4 and S5). Cylinder labeling is due to PG synthesis as the dipeptide ADA-DA was used. Incorporation for 2 or 10 min delineated the outline of the cylinder with labeling along its length (Figure S5), without revealing a distinct pattern. However, both SIM and STORM with FDAA labeling times of 15 s demonstrate a characteristic irregular banding or striped pattern (Figure 3A–C, Figures S4 and S5). Deciphering this arrangement is made more complex in that the fluorescence signal from a collapsed cylinder is made from two layers of cell wall material. The banding for double layer fragments has a broad distribution of interstripe distances (found using autocorrelation; see Methods) with a peak of around 360 nm (mean = 400 nm, SD = 150, *n* = 59) for SIM and around 180 nm for STORM (mean = 200 nm, SD = 110, *n* = 191) (Figure 3E,F). To get a more accurate spacing between the bands, SIM images of single layers were analyzed separately, and this gave a sharper distribution with a peak at 340 nm (mean = 310 nm, SD = 80, *n* = 18) (Figure 3G). The discrepancy between the SIM and STORM data and the broad distribution of the SIM distances in the double layers may occur due to the overlay of the two sides of the cell and the relatively lower resolution of SIM, with SIM tending to blur the stripe intensities from the two sides of the septum into a single broad peak.

The question then arises as to how this peptidoglycan insertion pattern maps onto known cylinder architecture? We have recently shown that the cylinder is characterized by an open mesh at the outer surface, while the inner surface where synthesis takes place has a tight mesh.^3^ This inner mesh encompasses glycan strands which are loosely oriented parallel to the short axis of the cell and at larger length scales form bands of material that we have previously termed “cables”.^23^ In some cases, sacculi break such that a single leaflet of cell wall is adhered to the surface (Figure 3C, white box). SIMForce of this peptidoglycan material, with the internal (plasma membrane facing) side exposed for AFM imaging, clearly identifiable as the internal surface due to the different architectures of the two sides,^3^ indicates that the areas of recent synthesis are generally slightly thicker than the surrounding material and that the wall appears denser in these regions (Figure 3D).

The implication of these data is that synthesis in the rod occurs in circumferential bands/stripes, corresponding to the “cables”. MreB and affiliate proteins have previously been correlated with the Rod complex associated cell wall synthesis.^24,21^ Both MreB and cell wall synthesis machinery show coupled dynamics that produce the circumferential motion of the Rod complex.^25,9,21^ Therefore, we hypothesized that MreB and MreB-like proteins may be essential for the striped insertion of new peptidoglycan material. To explore this, we used a strain deficient in MreB and its two orthologs, Mbl and MreBH (4577, *Ωneo3427 ΔmreB Δmbl::cat ΔmreBH::erm Ω(neo::spc ΔrsgI*).^26^ The parental strain (4624 *ΔrsgI::(neo::spc)*) showed no difference from the HR168 strain used throughout (data not shown). The *ΔmreB Δmbl ΔmreBH* cells were greatly enlarged compared to wild-type and displayed a spherical phenotype as previously published.^26^ 15 s of ADA-DA labeling demonstrated a patchy insertion pattern all over the cell wall, with no stripes and no significant periodicity (Figure 3H). AFM analysis of the sacculi similarly revealed a patchy surface architecture. SIMForce again largely correlated denser and slightly raised regions of the cell wall with areas of most recent peptidoglycan synthesis (Figure 3I). From this, it is apparent that the orientation of recent synthesis into stripes is associated with the presence of the MreB family of synthesis guiding proteins and also with the formation of rod-shaped cells.

## Conclusions

The data presented here, building on our recent high-resolution studies of peptidoglycan,^3^ provide a molecular framework to understand the process of cell wall synthesis within its architectural context, for the model Gram-positive rod-shaped organism *B. subtilis*. During septation, initial peptidoglycan synthesis occurs through the deposition of tight concentric rings on what will become the outer pole surface of the daughter cell (“rings”).^3,23^ Immediately behind this leading edge there is rapid synthesis of the majority of the thickness of the cell wall which consists of approximately randomly oriented peptidoglycan. This rapid synthesis leaves a large number of “pores” behind and the cytoplasm facing side of the wall incomplete. Subsequent synthesis over the entirety of the septal surface fills in these pores and adds additional material to the surface of the septum, resulting in the final dense mesh seen on completed poles. During cell elongation, synthesis occurs in rough bands of material, approximately 310 nm apart, directed by the MreB family of proteins.

Correlation of high-resolution AFM with super-resolution optical microscopy, on the same sample, in the same image, has provided otherwise inaccessible insights into how the cell wall is synthesized. The wider application of our approach can begin to elucidate cell wall synthesis in other systems such as fungi and plants.

## Methods

### Bacterial Strains and Growth Conditions

*Bacillus subtilis* (HR168) were grown on nutrient agar (NA) or nutrient broth (NB) at 37 °C with aeration at 250 rpm. Strains 4264 (*rgsI::spc*) and 4277 (*Ωneo3427 ΔmreB Δmbl::cat ΔmreBH::erm Ω(neo::spc) ΔrsgI*) were selected with appropriate antibiotics on solid media and grown without antibiotics in liquid culture. 4277 was cultured in the presence of 20 mM MgSO_4_, as it is strictly Mg^2+^ dependent. When required, antibiotics were added at the following concentrations: spectinomycin (50 μg mL^–1^), chloramphenicol (5 μg mL^–1^), erythromycin (1 μg mL^–1^), and kanamycin (5 μg mL^–1^).

### Preparation of Labeled *B. subtilis* Sacculi

*B. subtilis* cells at midexponential phase were incubated with ADA (1 mM; Iris Biotech) or ADA-DA^13^ (1 mM) for 15 s or 2 or 10 min prior to rapid boiling to kill bacterial cells and inactivate any potential hydrolase activity. Cells were broken by FastPrep or French Press, then suspended in 5% (w/v) SDS, and boiled for 25 min, and the sacculi were collected by centrifugation at 20,000*g* for 3 min. The resulting pellets were washed with distilled water to remove all traces of SDS, then resuspended in Tris-HCl (50 mM, pH7) containing 2 mg mL^–1^ Pronase and incubated at 60 °C for 90 min. The resulting sacculi were then resuspended in LC-MS Chromasolv water for storage at −20 °C.

Alkyl fluorophores (AlexaFluo647 or Atto488) were attached to ADA/ADA-DA-labeled sacculi using the Click-iT kit (Invitrogen). Click-iT reaction buffer was made using the manufacturer’s instructions containing alkyne dye at 10 μg mL^–1^. This reaction either occurred within an Eppendorf tube where *B. subtilis* sacculi were resuspended in 500 μL of Click-iT reaction buffer or on a coverslip where sacculi dried onto a glass coverslip were flooded with Click-iT reaction buffer. In both cases, the reaction was allowed to continue for 30 min at room temperature before being washed with Milli-Q water.

### Sample Preparation for Microscopy

Glass coverslips (high-precision, no. 1.5H, 22 × 22 mm, 170 ± 5 μm, Marienfeld) or glass grids (28 mm diameter, no. 1.5H (170 μm ± 5 μm) D 263 Schott glass, 50 μm grid) were sonicated for 15 min in 1 M KOH and washed with water. These cleaned coverslips were either incubated with poly-l-lysine solution (0.1% (w/v)) or Cell-Tak to ensure attachment of sacculi on the glass surface. Sacculi were diluted in HPLC-grade water to appropriate concentration and dried onto glass coverslip/grid using N_2_. These were further washed and dried with N_2_ again to remove any unattached sample.

### Structured Illumination Microscopy

For wide-field and SIM imaging, coverslips were mounted onto glass slides with PBS as the imaging buffer. Wide-field deconvolution and SIM were carried out using a v4 DeltaVision OMX 3D-SIM system fitted with a Blaze module (Applied Precision, GE Healthcare, Issaquah, USA). For wide-field imaging, samples were illuminated using a six color solid-state illuminator (LED). The objective was a 60× NA 1.42 oil plan apochromatic lens, and the system had a standard BGR filter set and used a scientific CMOS camera. For 3D-SIM, samples were illuminated using laser illumination. For each *z* slice, samples were imaged in 5 phase shifts and 3 angles, and *z*-steps were 0.125 μm. Reconstructions were performed with the Softworx software (GE Healthcare) using optical transfer functions (OTFs) optimized for the specific wavelength and oil used. The same software was used for deconvolution.

### Transmission Electron Microscopy

*B. subtilis* was grown to mid-exponential phase (OD_600_ ∼ 0.4), cells were collected by centrifugation, and pellets were chemically fixed with 2.5% glutaraldehyde EM grade in PBS overnight at 4 °C. The pellet was further washed three times in buffer and incubated with 2% aqueous osmium tetroxide for 2 h. After two buffer washes, the specimens were dehydrated in a series of increasing concentrations of ethanol (75%, 95%, 100%, and 100% dried ethanol), followed by two 15 min incubations with pure propylene oxide. Specimens were embedded in Epon resin, and 80 nm thin-sections were mounted on 200-square mesh copper TEM grids and stained with 3% w/v uranyl acetate and Reynold’s lead citrate.^27^ Images were obtained in a FEI Tecnai T12 Spirit transmission electron microscope operating at 80 kV. Images were recorded on a Gatan ORIUS SC1000B bottom-mounted CCD camera.

### NSTORM and Reconstruction

For STORM, coverslips were mounted onto glass slides with GLOX (0.5 mg mL^–1^ glucose oxidase, 40 μg mL^–1^ catalase, 10% (w/v) glucose in 50 mM Tris-HCl containing 10 mM NaCl (pH 8.0)) containing 100 mM mercaptoethylamine (MEA) as the imaging buffer.

Localization microscopy was carried out using a Nikon Ti-NS N-STORM version 1 in continuous mode. The objective used was an SR Apo TIRF 100× NA 1.49, and images were detected using EMCCD camera (Andor DU-897) using the 17 MHz 16-bit mode with an EM Multiplier Gain of 300 and a conversion gain of 3. Atto 488 was imaged using a Coherent Sapphire 488 nm 200 mW laser, while AlexaFluor647 was imaged using the 650 nm laser. Imaging was carried out under oblique illumination but not full TIRF. Images were reconstructed using the Nikon Elements software, with further drift correction applied *via* cross-correlation of localizations in ThunderSTORM, an ImageJ/Fiji plugin.^28^

### STORMForce

A 100× oil immersion objective with 1.42 NA and a charge coupled device (CCD) Hamamatsu camera was added to a Nikon Ti Eclipse inverted stereomicroscope base. A JPK Nanowizard III Bio was placed on top of the microscope body. STORM and AFM data were collected sequentially, always STORM first in the data presented here. Some experiments were performed with AFM data collected first to confirm that the order did not impact on the results obtained. AFM data collecting is considerably more time-consuming than STORM, so imaging with AFM after STORM avoids inefficiencies if the STORM fails for some reason. Initially TetraSpeck fluorescent microspheres (ThermoFisher Scientific) were used to provide fiducial markers between AFM and STORM images. However, it was found that these were not necessary, and image overlay could be performed by a combination of dead-reckoning and image adjustment due to the large number of clear features in the STORM and AFM images obtained, so the microspheres were not incorporated in the workflow for the data shown.

RS Imaging software by Photometrics was used to collect the STORM data, adjust the threshold, and set an acquisition speed to 31 fps. STORM images were reconstructed and rendered using ThunderSTORM, an ImageJ/Fiji plugin.^28^

All AFM data were taken in quantitative imaging mode in HPLC grade water using a FastScanD cantilever, nominal spring constant 0.25 N m^–1^ with a 256 × 256 pixel scan region. AFM images of septa in Figure 2 and Figure S2 were first order flattened and high pass filtered with a filter size of 0.20 μm. AFM images in Figure 3 where first order flattened, and all AFM images were processed using JPK data processing software (version spm-6.1.4.9).

For integrated STORMForce, an open to the air sample preparation was used. A glass coverslip was placed inside a small Petri dish with a hole cut out of the bottom and flooded with GLOX imaging buffer (as for NSTORM). For subsequent AFM imaging, the GLOX buffer was replaced with HPLC grade water.

### SIMForce Grid Samples

Sacculi on gridded coverslips were mounted and sealed onto glass slides with PBS for SIM data acquisition. Coverslips were then removed from the slides, washed, and imaged using AFM.

### Analysis of Localization Microscopy Data

Septal localizations were selected from fields and analyzed as in ref^13^ except that localizations were fitted to a circle throughout.

### Autocorrelation of Striped Insertion

Fourteen pixel (SIM) or 17 pixel (STORM) wide profiles were taken along the long axis of the cell on isolated sacculi. The profiles were then resampled with a sampling period of 20 nm (so that the SIM and STORM data can be compared). The new resampled profile was then normalized by subtracting the mean of the profile to every data point in the profile. The normalized profile was then autocorrelated using the ‘xcorr’ function in MATLAB. The peaks in the autocorrelation intensity profile were found using the ‘findpeaks’ function in MATLAB using the default settings. The distances between these peaks were then computed by finding the difference in the peak positions. The distances from all the profiles from the same experiment were combined, and histograms were plotted with bin size of 50 nm.

For sacculi that were only a single wall thick, the profile was calculated using a 28 pixel wide profile to improve the signal-to-noise ratio. The rest of the data analysis was the same as above.

### Analysis of Pore Size

Analysis was completed using ImageJ/Fiji. First, AFM images of septa were sectioned into 50 nm increments from the leading edge. Then a threshold was applied to highlight the pores, and the Analyze Particles plugin was used to produce a map of the pores and measurement of pore and nonpore area and number of pores.

### Access to data

All data presented in this paper are available through figshare at 10.15131/shef.data.16629064. Sources of materials and analysis software are described in the Methods section. Any additional information that may be required is available from the authors upon request.

## Supporting Information Available

Figure S1: *B. subtilis* septal synthesis labeled with FDAAs imaged with SIM. Figure S2: *B subtilis* septal synthesis STORMForce images. Figure S3: TEM images and analysis of stages in septal growth. Figure S4: SIM images of cylinder peptidoglycan synthesis in *B. subtilis*. Figure S5: STORM images of cylinder peptidoglycan synthesis in *B. subtilis* (PDF)
