# Supplementary material for: Correlative Super-Resolution Optical and Atomic Force Microscopy Reveals Relationships Between Bacterial Cell Wall Architecture and Synthesis in Bacillus subtilis
Source: ACS Nano. 2021 Sep 17;15(10):16011–8. doi: 10.1021/acsnano.1c04375 (PMC8552488; doi:10.1021/acsnano.1c04375)
Supplement: Supplementary file 1 — nn1c04375_si_001.pdf [file nn1c04375_si_001.pdf]

Supporting information

Correlative Super-Resolution Optical and Atomic Force Microscopy Reveals Relationships Between Bacterial Cell Wall Architecture and Synthesis in *Bacillus Subtilis*

Raveen K.G. Tank[1,2]*, Victoria A. Lund[3,4]*, Sandip Kumar[5], Robert D. Turner[3,4,6], Lucia Lafage[3,4], Laia Pasquina Lemonche[1,4], Per A. Bullough[3,4], Ashley Cadby[1], Simon J. Foster[3,4]†, Jamie K. Hobbs[1,4]†

*These authors contributed equally

†To whom correspondence should be addressed. E-mail: Jamie.Hobbs@sheffield.ac.uk, S.Foster@sheffield.ac.uk

[1]Department of Physics and Astronomy, University of Sheffield, Sheffield, S3 7RH, UK.

[2]Current address: School of Biological Sciences, University of Manchester, Manchester, M13 9PT, UK.

[3] Department of Molecular Biology and Biotechnology, University of Sheffield, Sheffield, S10 2TN, UK.

[4] The Florey Institute for Host-Pathogen Interactions, University of Sheffield, Sheffield, S10 2TN, UK

[5]Department of Biochemistry, University of Oxford, Oxford, OX1 3QU, UK.

[6]Department of Computer Science, University of Sheffield, Sheffield, S1 4DP, UK.

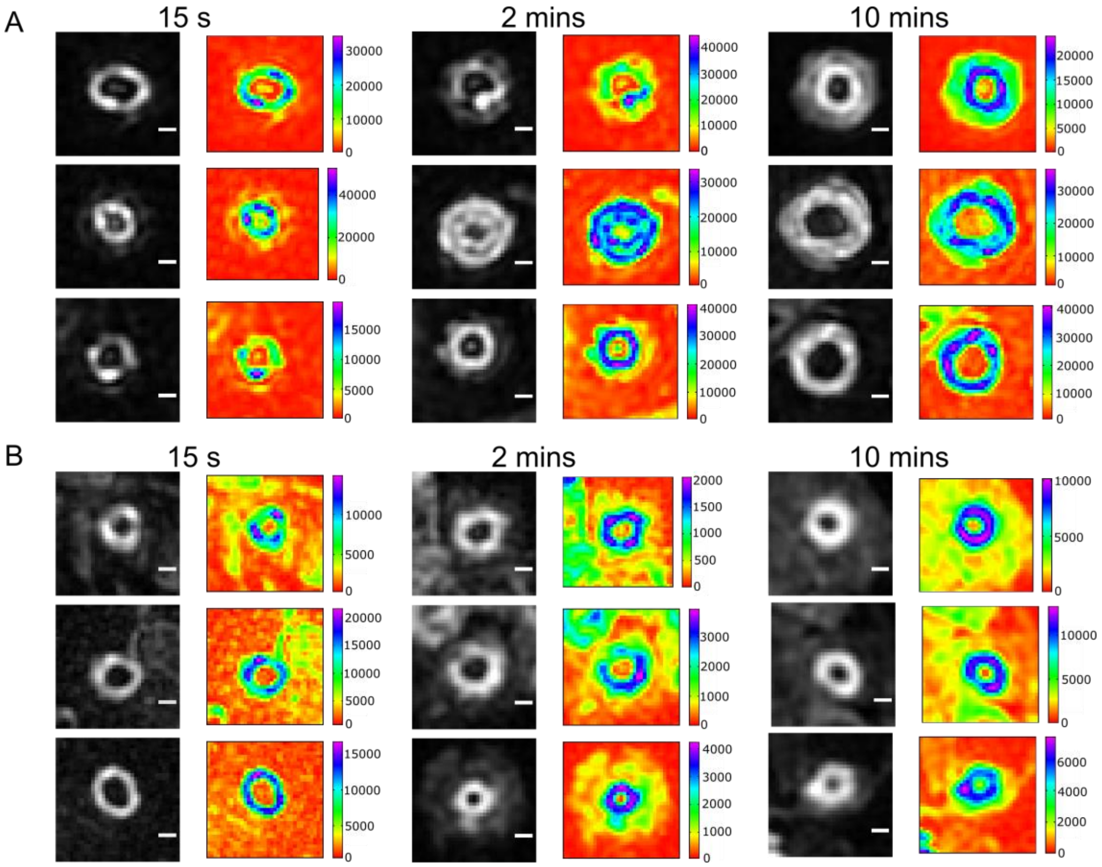

Supplementary Figure S1. Peptidoglycan synthesis in *B. subtilis* septa labelled with FDAAs for 15s, 2 min & 10 min and imaged using SIM. Images of representative septa (scale bar 200 nm) along with heat maps where violet on the colour scale represents areas of high fluorescence intensity and red shows low intensity. A) ADA labelling, B) ADA-DA labelling.

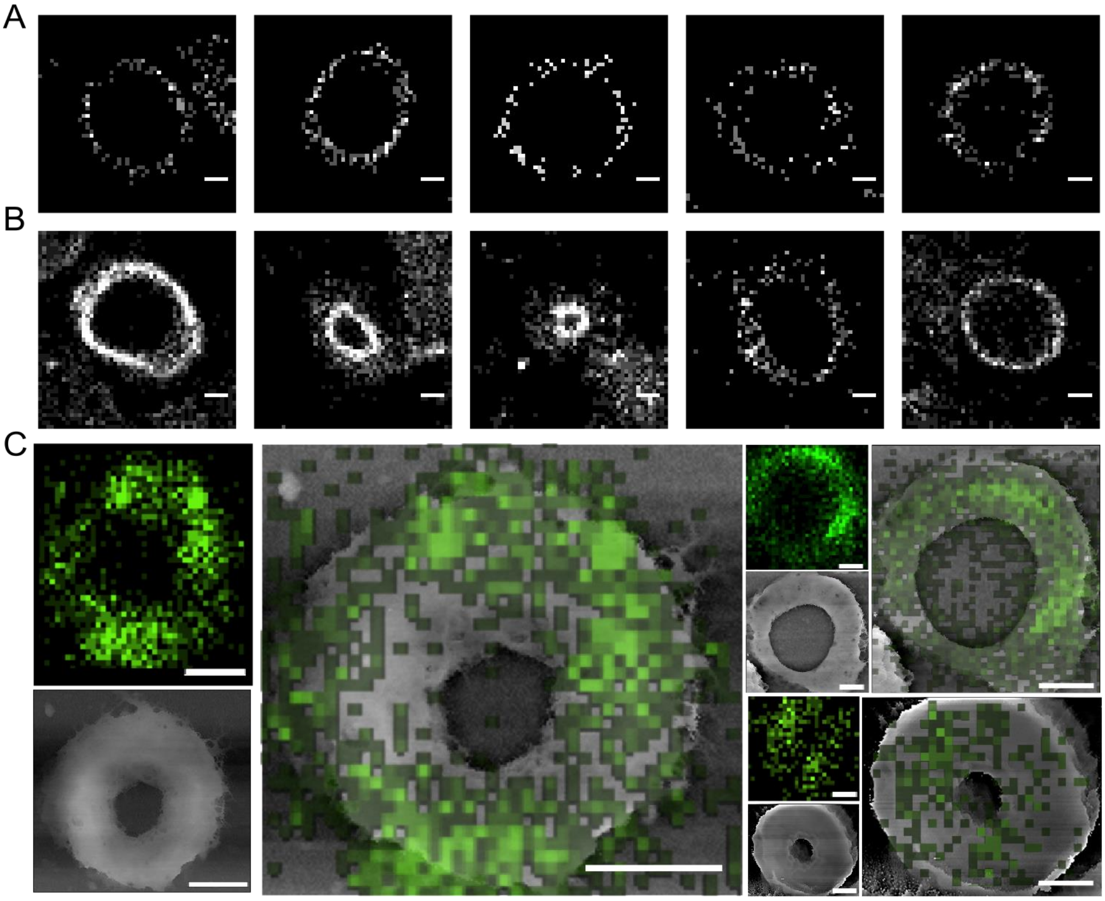

Supplementary Figure S2. Septal peptidoglycan synthesis in *B. subtilis*. A) 15s ADA-DA STORM images (scale bar 200 nm). B) 2 min ADA-DA STORM images (scale bar 200 nm). C) STORMForce imaging of 2 min ADA labelled septa showing high intensity localisations (scale bar 200 nm).

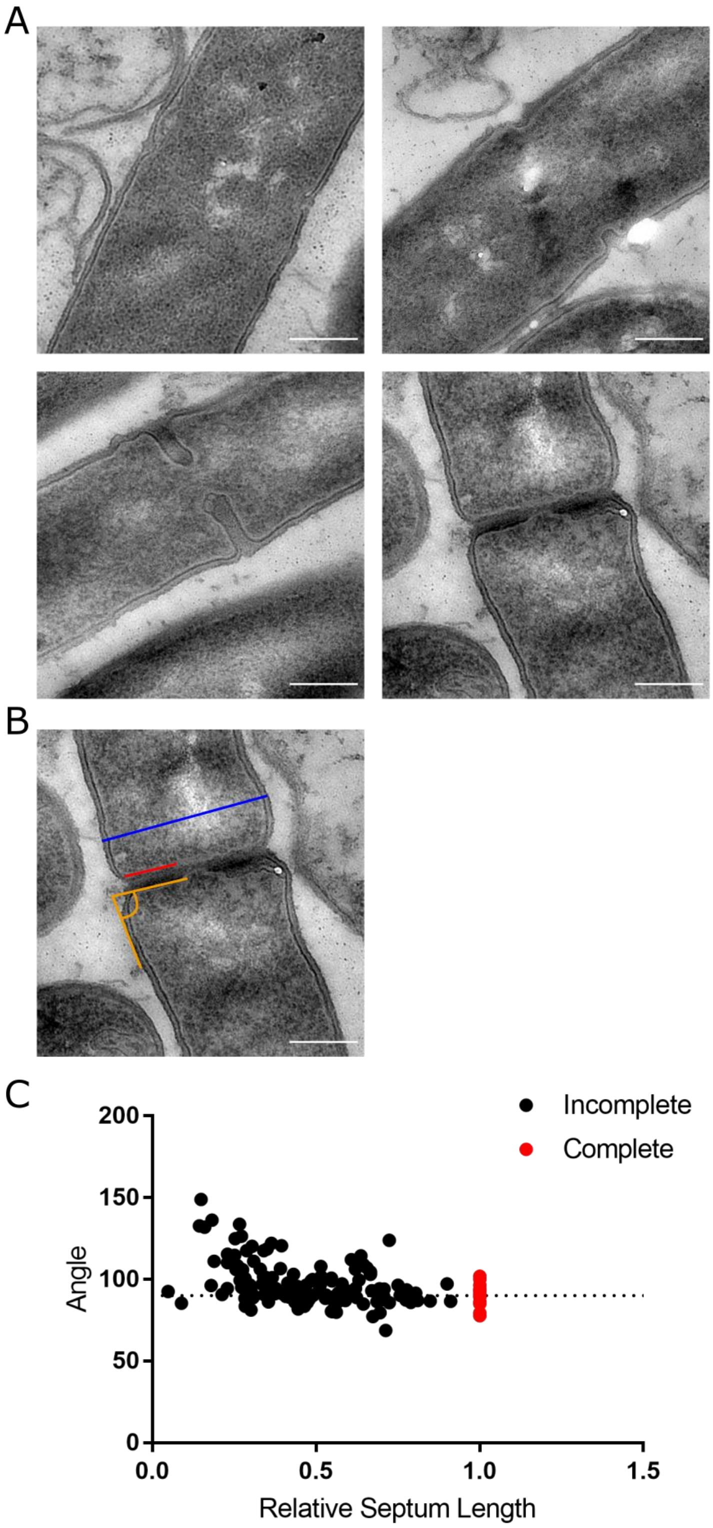

Supplementary Figure S3. A) Representative TEM Images of *B. subtilis* septa in whole cells (scale bars 250 nm). B) Annotation showing how septal angles to cell surface are measured. Red line is septum length, blue is the whole cell diameter and yellow is the angle measured. C) Measurement of the angle between a line parallel to the surface of the septum and a line parallel to the surface of the bacterium in incomplete (black) and complete (red) septa.

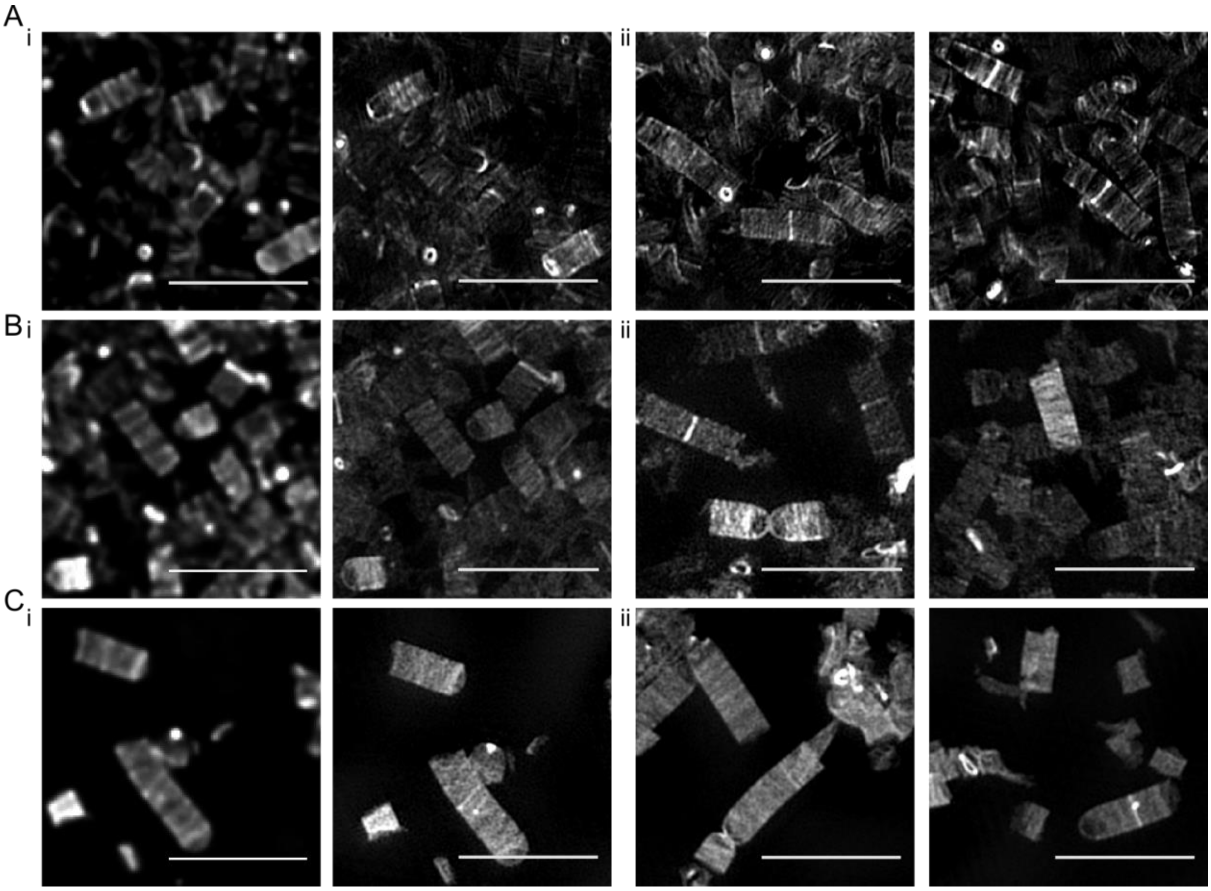

Supplementary Figure S4. Cylinder peptidoglycan synthesis in *B. subtilis*. A) 15s ADA-DA, B) 2 min ADA-DA, C) 10 min ADA-DA. i) Deconvolved and SIM image of the same area, showing lines are not a result of SIM reconstruction, ii) additional SIM images (scale bar 5 µm).

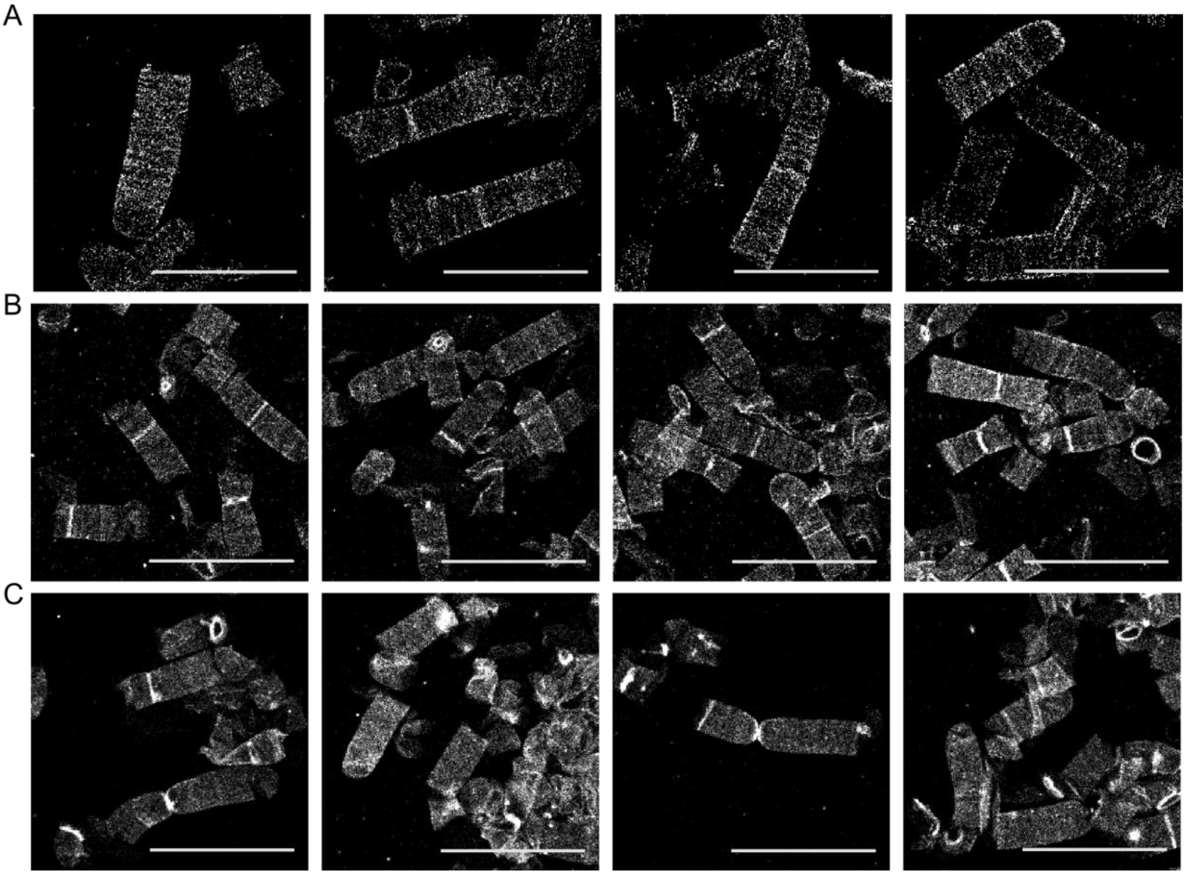

Supplementary Figure S5. Cylinder peptidoglycan synthesis in *B. subtilis*. A) 15s ADA-DA, B) 2 min ADA-DA, C) 10 min ADA-DA of STORM images showing lines also seen in supplementary figure S4 SIM images (scale bar 5 µm).
